# Adolescent school injuries and classroom sex compositions in German secondary schools

**DOI:** 10.1186/s12889-021-12370-8

**Published:** 2022-01-10

**Authors:** Andreas Filser, Sven Stadtmüller, Robert Lipp, Richard Preetz

**Affiliations:** 1 Institute for Employment Research (Institut für Arbeitsmarkt- und Berufsforschung, IAB), Nuremberg, Germany; 2 Research Centre of Demographic Change (FZDW), Frankfurt University of Applied Sciences (FRA-UAS), Frankfurt am Main, Germany; 3 SOCIUM Research Center on Inequality and Social Policy, University of Bremen, Bremen, Germany

**Keywords:** School injury, sex ratio, adolescent health, peer effects, sex composition

## Abstract

**Background:**

School injuries are an important adolescent health problem. Previous research suggests that relevant risk behaviors for school injuries, risk-taking and aggression, are highly susceptible to peer effects. Specifically, evidence suggests that the ratio of men and women in peer groups (sex ratio) affects individuals’ propensity for aggression and risk-taking. However, potential associations of classroom sex ratios with adolescent school injury risks have not been studied so far. The purpose of this paper is to investigate the association of classroom sex compositions with adolescent school injuries.

**Methods:**

We investigate the association of classroom sex ratios with school injuries in a longitudinal survey dataset containing 13,131 observations from 9,204 adolescent students (ages 13-16) from secondary schools in Germany. The data also allow us to identify injuries due to aggressive behavior and analyze these injuries in detail. We use multilevel logistic regression models to analyze risks of both overall and aggression-related school injuries.

**Results:**

Adolescent students’ risk for school injuries is significantly and positively associated with male-skewed classroom sex ratios (OR = 1.012, *p=0.012*). Specifically, the risk of sustaining a school injury increases by 33.5 percent when moving from the 10^th^ to the 90^th^ classroom sex ratio percentile. Moreover, we find an even stronger positive association between male-dominated classrooms and aggression-related injury risks (OR = 1.022, *p=0.010*). Compared to classroom sex ratios at the 10^th^ percentile, the risk of an aggression-related injury is 78 percent higher in classrooms with a sex ratio at the 90^th^ percentile. Finally, we find that both boys’ and girls’ injury risks equally increase with a higher proportion of male students in their classroom.

**Conclusions:**

Our findings indicate that sex composition of classrooms is an important contextual factor for adolescent school injuries, in particular school injuries resulting from aggression. These findings illustrate the need to integrate a contextual perspective on school injuries among adolescent students both into research and into intervention planning.

**Supplementary Information:**

The online version contains supplementary material available at 10.1186/s12889-021-12370-8.

## Background

School injuries are a key public health problem [1–5]. Injuries are the leading cause of adolescent mortality [6–9], and even non-fatal injuries have several detriments, such as temporary or permanent disabilities [3, 10–12]. These consequences entail a deterioration in well-being and an increase in school absenteeism, potentially lowering academic achievement of affected students [3, 13–15]. Studies reveal that about 25 percent of all injuries to children under the age of 17 occur at school [5, 9, 16–21]. For Germany, the social accident insurance reports that in 2019, a total of 1.17 million school injuries required medical treatment [1]. Adolescent students are particularly vulnerable to school injuries, compared to both older and younger students [3, 9, 20, 21].

Despite the prevalence and consequences of adolescent school injuries, knowledge on specific risk factors remains limited. In general, the literature attributes school injuries among adolescents to the increase in risk-taking and violent behavior during adolescence [9, 22, 23] These crucial risk behaviors for adolescent school injuries are closely associated with peer effects [24–30]. However, peer contexts have rarely been considered in the literature on school injuries so far. Previous studies have relied on medical record data to investigate patterns among injured students [3, 5, 9, 12, 20, 31]. However, injury records lack information on non-injured counterfactuals, inhibiting analyses of factors that might foster or prevent school injuries. A few studies have analyzed data on both injured and non-injured students, but focused on selected individual-level risk factors or the physical environment at schools [4, 23, 32, 33].

In this paper, we focus on the potential role of classroom sex compositions for adolescent school injuries to add a contextual perspective on school injuries. A contextual perspective on adolescent school injuries is essential because both key risk factors – risk-taking and aggression – are largely a group phenomenon. Adolescents spend more time with their peers and exhibit the highest level of concern with being accepted in relevant peer contexts than any other age group [34, 35]. The crucial role of peer contexts for adolescents particularly concerns effects on risk-taking and aggression, since both behaviors disproportionately occur when adolescents are with their peers [36, 37]. An extensive body of literature documents that peer norms and behavior patterns of peers are a primary contextual factor for adolescent risk-taking, violence, and aggression [27–29, 34, 37–40]. Classmates constitute important peer contexts because adolescent students spend around 50% of their waking hours in school [41]. Classroom norms and social ties within these contexts create peer pressure towards or away from risky or aggressive behavior, and adolescents are particularly susceptible to these peer group dynamics: adolescents, particularly boys, are more likely to engage in risky behavior and aggression when classroom peers display such behavior [25, 42]. Thus, given the importance of risk-taking and aggression for adolescent school injuries, classroom peer contexts constitute a crucial aspect in order to arrive at a better understanding of school injury risks.

A key source of contextual peer effects on adolescent school injury risks might be the sex composition of a classroom, i.e. the sex ratio of boys and girls in a class. Evidence from experiments and observational data suggests that aggression and risk-taking are associated with the sex ratio in a given context [43–50]. The literature identifies male status thriving and competitive orientation as key sources for levels of physical aggression by men, including male adolescents [51–53]. Adolescent boys exhibit a stronger orientation towards physical competition and domination than girls. Male adolescents are also more likely to consider physical aggression to be an effective means of attaining social status among peers [24, 26, 51]. Empirical evidence suggests that this male tendency for physical rivalry is exacerbated in male-dominated contexts, resulting in higher levels of violence, particularly against other men [43, 47, 48, 54–57]. For adolescents, previous research has demonstrated that peer effects on adolescent delinquency are contingent on the sex constellations of peers [26, 30]. Moreover, higher levels of violence mediate the negative relationship between classroom shares of male students and academic performance in classes with high shares of male students [58]. Similarly, exposure to female in-school peer contacts decreases adolescent males’ odds of engaging in serious violence, while having more male friends is associated with increased violence by girls [24]. Evidence for sex ratio effects on risk-taking also exists. For instance, male-dominated contexts appear to instigate risk-taking in experimental [44, 45, 59] and health-risky behavior in non-experimental studies [49, 60].

This paper explores whether and how classroom sex ratios are related to school injury risks. Sex ratios have been shown to correlate with behavioral patterns that are relevant for school injury risks, however, the role of classroom sex constellations for school injury risks has not been explored yet. To examine this relationship, we use data from a large-scale longitudinal survey study on school injuries and health of adolescents in Germany [61]. The results provide policy makers, school officials, and other stakeholders seeking to reduce violence and injuries in schools with a perspective that may offer ways in dealing these issues beyond individual interventions.

## Material and Methods

### Data and study population

Data for our study come from the German study on Health Behavior and Injuries in School Age (GUS, www.fzdw.de/gus), a large-scale panel survey of children and adolescents funded by the German Social Accident Insurance (DGUV - *Deutsche Gesetzliche Unfallversicherung*). GUS collected data in 14 of 16 German federal states, and the study comprises information on 18,365 students from 173 schools, surveyed at least once during their secondary education between 2014 and 2019.

At each participating school, GUS surveyed all students in the respective grade. The data was collected using a computer-assisted self-administered classroom survey, for which each student received a tablet device to answer the questionnaire during a (regular) school period (of 45 minutes). Trained interviewers introduced the survey, explained the handling of the devices, and helped students with potential technical or other problems.

We limit our analytical sample to adolescent age stages by focusing on students in the 8^th^ and 9^th^ grades. Consequently, students in our dataset are aged 13-15 (8^th^ grade) and 14-16 (9^th^ grade) in the 2017/18 and 2018/19 school years, respectively. Limiting our sample to data from 8^th^ and 9^th^ grade students ensures a close match between class compositions at the time of the survey and the injury. This is because GUS surveyed injuries retrospectively, i.e. students reported injuries from the past 12 months. Therefore, students might have reported injuries from the previous school year, given that the interviews mostly took place around the halfway mark of the school year. However, in many federal states, schools rearrange class compositions based on students’ choices of major subjects before 7^th^ grade. Consequently, injuries reported in the 7^th^ grade panel wave might date back to 6^th^ grade, where students were exposed to a different class composition. We circumvent this problem by excluding data for the 7^th^ grade. Moreover, we focus on the adolescent age stages because research has demonstrated the extraordinary importance of peer constellations in this life stage [25, 28, 34, 37, 39, 40, 42]. At the same time, adolescence correlates with increased risks for school injuries due to heightened propensities for aggressive behavior at this life stage.

### Measures

#### Dependent variable

Our dependent variable captures whether students reported sustaining an injury in the school environment within the last 12 months. The GUS questionnaire also asked students who reported a school injury to specify the context of this injury (i.e., whether it happened during physical education, on the schoolyard, in the school building, or on the way to school). In case of multiple injuries, the questionnaire instructed students to report details on the injury with the longest recovery time.

Our analysis focuses on injuries that took place on the schoolyard or in the school building. We expect these injuries to be related with classroom sex ratios because, in Germany, students spend most of their day together with the same group of students. In contrast, physical education often takes place in sex-specific groups of students from different classes. Therefore, we exclude injuries that occurred during physical education from our analysis to avoid mismatches between general class compositions and student group constellations at the time of the injury. Similarly, we exclude injuries occurring on the students’ way to school because these injuries took place outside the school- and class context.

Since we expect injuries resulting from aggression to be particularly related to classroom sex ratios, we specifically focus on aggression-related injuries. Using a five-point Likert-scale, students were asked (1) whether someone else was responsible for this injury and (2) whether this injury resulted from their own fault. We categorize injuries as aggression-related if students attributed their injury to a third person while rejecting the statement that the injury was their own fault.

#### Classroom sex ratio

Our key independent variable is the classroom sex ratio, operationalized as the share of male students in a class. Students in the 8^th^ and 9^th^ grade in Germany typically spend the majority of their school day as a class with the same group of students. Therefore, classroom sex ratios capture the composition of the students’ main peer group at school.

We operationalized the sex ratio as the classroom share of male students ranging from 0 to 100 percent in order to avoid the asymmetrical distribution of proper ratios [62]. Thus, a classroom sex ratio of 50 percent indicates an equal number of male and female students. We estimate classroom sex ratios based on respondents from each class. In order to ensure close approximations of the true classroom sex ratio, we limit our analytical sample to only those classes in which at least ten students participated in the survey. We do not find an indication for classroom sex ratios to be correlated with participation rates on the class level (Pearson’s r = .001, *p=.764*, Figure S1 in Supplementary Material 1). Nevertheless, we check the robustness of our results against the sensitivity to outliers or sex ratios with a high potential of bias due to a considerable level of non-response by fitting our models to a number of sample variants. These three sample variants either exclude (1) girls- and boys-only classes, (2) classes with sex ratios below 10 percent or above 90 percent, or (3) classes with a class-level response rate below 80 percent. Contrasting the results based on these subsamples with those from our main sample helps us narrowing down the problem of misrepresented sex ratios from potentially sex-selective non-response. Results from the auxiliary models can be found in Supplementary Material 2.

#### Control variables

Our data enable us to adjust our estimates for a set of potential individual- and aggregate-level confounders of adolescent school injuries. On the individual level, we include sex, migrant background (based on parental country of birth), family affluence, and a mental health index as controls in our models.

We measure parental socioeconomic status with the family affluence scale (FAS) proposed by Currie et al. [63]. The FAS has been validated at both national and international levels and shown to have good criterion validity regarding socioeconomic status (SES) and various health outcomes. FAS is less affected by non-response and recall error than SES measures that rely on children’s reports of household income or parental occupation [63]. Information on students’ mental health comes from eight items in which students were asked on how many days during the last week they (1) were irritated and in a bad mood, (2) felt fit and comfortable, (3) were full of energy, (4) felt sad, (5) felt lonely, (6) slept badly, (7) had problems concentrating, and (8) felt unhappy and depressed (α=.85).

On the class level, we integrate controls for the class mean of family affluence and the number of students in each class. We standardize the class mean of family affluence based on the wave-specific means and standard deviations (on the class level). Moreover, we control for differences between schools by integrating school type (with the Gymnasium as the most advanced school type in secondary education vs. all other school types), region (East vs. West Germany), and urbanity (with schools located in cities with more than 100,000 inhabitants coded as urban) as controls. Finally, we adjust for time trends using a dummy variable for the two panel waves in our data.

Table 1 displays the distribution of all variables included in the statistical models for our analytical sample. Students with missing values were deleted listwise. Values for the indexes of risk-seeking behavior, mental health status, and family affluence were only calculated if students responded to at least half of the items used for index construction. In total, our main analyses rely on two panel waves comprising 13,131 observations from 9,204 students in 520 classes at 132 schools.

**Table 1:** Sample characteristics. Students from 8^th^ and 9^th^ grade, GUS data 2018-2019

**Dependent variables**					
	% of obs. with school injury	4.4			
	% of obs. with school injury due to aggression	1.3			
**Continuous independent variables**		**mean**	**sd**	**min**	**max**
	Mental health (individual-level)	2.87	.75	0	4
	Family affluence (individual-level)	3.05	.72	0	4
	Family affluence (class-level, z-standardized)	0	1	-4.73	2.08
	Classroom sex ratio (class-level, percentage male students)	46.5	14.2	0	100
	Class size (number of students usually in class)	26.2	3.1	15	33
**Dichotomous independent variables**					
	% of obs. from male students	46.2			
	% of obs. from students with migrant background	30.6			
	% of obs. from students at the Gymnasium (upper secondary)	60.4			
	% of obs. from students at East German schools	15.3			
	% of obs. from students at rural schools	76.5			
	% of obs. from grade 9	45.6			
**Number of observations**		13,131			
**Number of students**		9,204			
**Number of classes**		520			
**Number of schools**		132			

### Analytical approach

Since we have a dichotomous dependent variable and hierarchical data (observations nested in students, and students nested in classes), we estimate multilevel logistic regression models to analyze the effect of classroom sex ratios on the individual likelihood to suffer an injury that took place either on the schoolyard or in the school building. Including random intercepts for students and classrooms allows us to adjust for time-invariant unobserved characteristics of school classes and students, respectively [64]. We also estimated models including a random intercept for schools as a fourth level but did not include them here since they essentially yield the same results.

Our main analysis consists of two parts. In the first part, we analyze the association of classroom sex ratios with school injuries on the schoolyard or in the school building. A second model series focuses only on injuries on the schoolyard or in the school building that resulted from third-party aggression. For both analyses, we report the results from the logistic regression models as odds ratios. Moreover, we transform the output from the logistic regression model to marginal effects. We then scale the differences in the estimated probability of the outcome by the baseline probability of experiencing the respective type of school injury. Therefore, our graphs show the percentage difference in the probability of the outcome at different levels of the sex ratio variable relative to the mean probability of the outcome in the analytical sample. Additionally, we report the relative risk of experiencing the respective type of school injury at the 10^th^ and 90^th^ percentile of the sex ratio distribution in our sample, which corresponds to a 29.4 (10^th^ percentile) and 63.6 percent (90^th^ percentile) share of male students. Finally, we test whether the association between the classroom sex ratio and injury risks differs between male and female students by integrating an interaction term for the individual’s sex and the sex ratio as well as a random slope for sex into our model [65, 66]. The Stata code for our analysis can be found in the supplementary material (Supplement 3).

## Results

### Any-cause school injury risks and classroom sex ratios

**Fig. 1:**
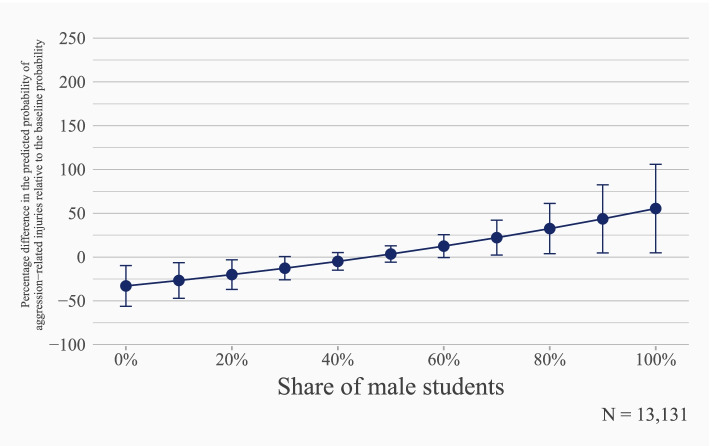
Adjusted predictions for school injury from multilevel logistic regression. Students from 8^th^ and 9^th^ grade, GUS data 2018-2019

In a first model, we analyze the association of classroom sex ratios with reports of any injury on the school premises. Results from a bivariate multilevel logistic regression model reveal a positive and statistically significant association between school injury risks and classroom sex ratios (OR = 1.017, *p<0.001*, see Table S1 & Fig. S2 in Supplementary Material 1). Multivariate results support this result: We find a positive and statistically significant association between classroom sex ratios and students’ susceptibility for school injuries (OR = 1.012, *p=0.012*, see Table S2 in Supplementary Material 1). Figure 1 illustrates that, despite the small odds ratio coefficient, the effect estimate for classroom sex ratios is far from negligible. In fact, the small odds ratio is mainly due to the scaling of the sex ratio to a range between 0 and 100 percent share of male students in a class. Therefore, the odds ratios reflect the change in injury risk for an increase in the share of male students by one percentage point. Figure 1 reveals a substantial variation in the percentage differences in the probability of sustaining an injury relative to the baseline probability across the range of the sex ratio. This is also apparent when comparing predicted injury risks. For instance, for classes in which 29.4 percent of the students are boys (the 10^th^ percentile), the model predicts a mean probability of injury on the school premises of 3.83 percent. In contrast, for classes with a 63.6 percent share of male students (the 90^th^ percentile), the respective value is 5.11 percent. In other words, the risk of a school injury increases by 33.5 percent when moving from the 10^th^ to the 90^th^ classroom sex ratio percentile.

In a complementary model, we test whether boys’ and girls’ injury risks are differently associated with classroom sex ratios. Thus, we also test whether the association between classroom sex ratios and injury risk varies between boys and girls. However, Table S3 and Figure S4 reveal that this is not the case (in Supplementary Material 1). We do not find a significant coefficient for the interaction term for individual sex and classroom sex ratios. In sum, this indicates that both male and female students similarly experience increased injury risks in classes with a higher share of male students.

### Aggression-related school injuries and classroom sex ratios

In our second series of analyses, we focus on aggression-related school injuries. Bivariate results again suggest a positive and statistically significant association of classroom sex ratios with school injuries from aggression (Table S4 and Fig. S4, in Supplementary Material 1). This association persists even after adjusting for individual- and contextual-level control variables: classroom sex ratios correlate significantly and positively with school injuries from aggression (OR = 1.022, *p=0.010*, Table S5 in Supplementary Material 1). Note that the odds ratio coefficient for the classroom sex ratio is higher for these aggression-related injuries than for all reported injuries on the school premises (OR = 1.012, see Table S2 in Supplementary Material 1).

**Fig. 2:**
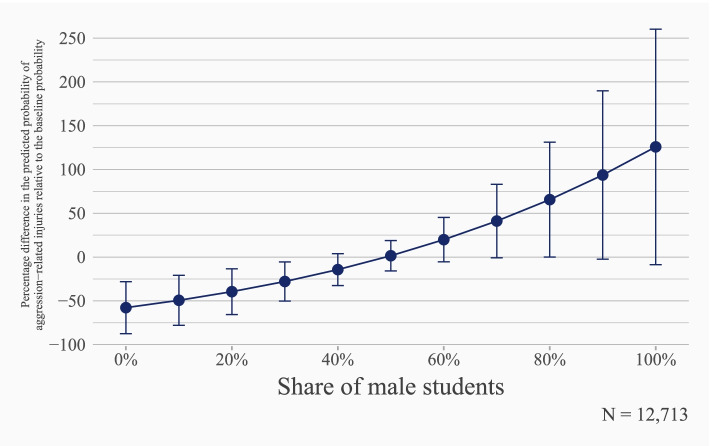
Adjusted predictions for aggression-related school injury from multilevel logistic regression. Students from 8th and 9th grade, GUS data 2018-2019

Figure 2 displays this steeper slope for the relative risk of aggression-related injuries compared to the slope from the model for all injuries. In particular, we find that the risk of school injuries from aggression increase more pronouncedly across the range of classroom sex ratios than any-cause school injury risks. This is also confirmed when we compare classes with a share of male students corresponding to the 10^th^ and the 90^th^ percentile, respectively. Here, the predicted risks are 0.91 and 1.62 percent, translating into a difference of 78 percent. For comparison, the equivalent increase for all injuries was 33.5 percent. Note, however, the considerably broader confidence intervals compared to Fig. 2. This is attributable to the lower mean risk of aggression-related injuries compared to all injuries (1.3 percent vs. 4.4 percent), as displayed in Table 1.

Finally, we do not find an indication that boys’ and girls’ risks of aggression-related injuries are differently related to the classroom sex ratio. Figure S5 and Table S6 display the results from regression models that included an interaction effect for sex and classroom sex ratios (in Supplementary Material 1). While there is a slight divergence in the slopes of predicted risk between boys and girls, this difference remains small across the entire range of the classroom sex ratios. In sum, our results suggest the risk of aggression-related injuries to increase as the share of male students gets larger – and this holds true for boys and girls alike.

### Robustness checks

Finally, auxiliary models confirm that our results are robust against a number of alternative sample definitions. Therefore, we rule out that our findings might be due to a few outliers in terms of the classroom sex ratio. Specifically, the coefficient estimate for classroom sex ratios remains the same even when dropping classes with the most extreme sex ratios from the sample. Models fitted to subsamples that exclude single-sex classes or classes with a share of male students below 10 percent or above 90 percent suggest a positive association between classroom sex ratios and school injuries similar to our main results presented above. An additional model variant fitted to a sample of only those observations with at least 80 percent response rate on the class-level yields a comparable positive association. However, the coefficient is not significant due to the reduced number of cases in this model (Figures S2.1-S2.2 and Tables S2.1-S.2.2, Supplementary Material 2).

For the aggression-related injuries, models fitted to these alternative sample definitions also support our main findings. The coefficients and predicted relative risks are essentially the same across the sample specifications. Moreover, coefficients for the classroom sex ratio in models predicting aggression-related school injuries are larger than the corresponding estimate in the models for the overall school injuries. However, the reduced sample size turns the coefficient for the classroom sex ratios insignificant when limiting our sample to observations with a minimum class-level response rate of 80 percent. Nevertheless, the coefficient and predictions based on this restrictive sample definition support the overall finding of a positive association between aggression-related school injury risks and shares of male students in the classroom. The full results from these auxiliary models can be found in Supplementary Material 2 (Figures S2.3-S2.4 and Tables S2.3-S2.4).

## Discussion

In this paper, we use nationwide survey data from Germany to investigate the link between classroom sex ratios and adolescent school injuries. School injuries are a major public health problem with important implications for students’ quality of life and academic achievement [2–4, 10, 13–15, 17, 18]. However, empirical evidence on contextual risk factors for adolescent school injuries has been lacking so far. This is despite the fact that key risk factors for adolescent school injuries – aggression and risk-taking – have consistently shown to be highly susceptible to contextual influences. Moreover, a growing body of literature suggests that male-skewed sex ratios increase adolescent risk-taking and in particular physical aggression [24, 26, 30, 43, 44, 47–49, 54–59]. In this paper, we bring together these spheres of expertise to explore the role of classroom sex compositions as a contextual risk factor for adolescent school injuries.

Overall, our analysis reveals four main findings. First, we find that adolescents face higher risks of sustaining school injuries when they are part of a male-skewed classroom. Second, additional models suggest that boys’ and girls’ injury risks are similarly associated with the share of male students in a classroom. Third, the association between classroom sex ratios and injury risks is even more pronounced when we focus our analysis on only those injuries that are attributable to aggression by other students. Thus, students in male-skewed classroom contexts are more likely to report an aggression-related school injury. Fourth, both boys and girls are equally at higher risk of sustaining an aggression-related injury when they are part of a male-skewed classroom sex ratio.

Our findings illustrate the need to integrate a contextual perspective in research on school injuries, particularly among adolescents. Previous studies have emphasized the crucial role of school facilities, environmental factors, and individual characteristics of students for school injury risks [4, 33]. Our results offer a complementary perspective by highlighting the social context of injury risks. This is not to downplay environmental risk factors such as weather, yard facilities, or architectural aspects for school injuries. However, the way students interact with these facilities and with one another is crucial for how likely these hazards translate into injuries. Contextual peer effects are particularly relevant for adolescents, including adolescent risk-behavior and aggression [27–29, 34, 36–40]. Our results illustrate the specific relevance of the composition of peer contexts at school for adolescent school injuries.

Interventions to prevent adolescent school injuries should therefore also take into account the sex composition of classrooms. Ensuring a mix of boys and girls in classrooms could help reduce the number of school injuries. Moreover, interventions should target specifically male-dominated classes. The literature suggests that the increase in injury risks in male-skewed classrooms results from higher levels of risk-taking and aggression in male-skewed contexts [24, 30, 43, 45, 47, 48, 54]. Both risk-taking and aggression have an important signaling function since it often confers status, particularly to adolescent males. Therefore, successful interventions should aim to specifically provide adolescents with alternative, safer forms of status attainment. Specifically, interventions should guide adolescents towards alternative means of gaining social acceptance and respect, where adolescents can attain status without contests or engaging in antisocial behavior [67]. For instance, a recently proposed framework highlights how processes of learning and self-reflection helps to counteract the establishment of toxic masculinity norms related to antisocial behavior [68].

This paper uses nationwide survey data with self-reports on school injuries, and analyses of such data come with certain limitations. Compared to studies relying on medical records, our data provide crucial information on the classroom context and information on non-injured students. However, information on school injuries is collected retrospectively, entailing the risk of recall error or recall bias. However, given that the survey focuses on severe injuries that required medical treatment, we expect that most affected students will be able to recall such a memorable event. A second limitation is that the aggression-related school injuries might not distinctly distinguishable from injuries resulting from other causes. In certain instances, injuries might be attributable to both third-party aggression as well as own risk-taking. Given that we find a similar association for both, all injuries and aggression-related injuries can, however, be considered as indicative of an overall association of sex ratios and injury risks. Moreover, while we can reconstruct classroom sex ratios, our data do not contain information on how closely students from a class interact with each other. Network approaches to peer effects frequently use directed friendship relations where students nominate their friends to reconstruct peer contexts for each student [24, 26, 30, 40]. Such information would help to approximate closer the sex composition of students’ peers. Nevertheless, network-based approaches also suggest that not only close friends, but also more distal peers do affect behavior in profound ways [40]. Consequently, our results constitute a baseline for a combined measure of sex compositions in close and distant peers at school. Finally, we cannot fully avoid problems of unobserved heterogeneity between schools and classes from differential preferences of boys and girls with respect to STEM or liberal arts [69]. However, we are able to attenuate this problem by adjusting for time-invariant unobserved characteristics of school classes by including random intercepts at the class-level.

Finally, further research is needed to clarify the mechanisms behind the link between classroom sex ratios and injury risks from risk-taking and aggression. Generally, the literature either draws upon biological differences between the sexes or gender-specific social expectations to explain sex ratio effects in behavior [70, 71]. While these approaches differ in their theoretical frameworks, both identify male status thriving and competitive orientation as prime sources for higher male involvement in physical aggression and health-detrimental risk-taking [51–53]. However, we acknowledge that we are not able to adjudicate on such pathways based on our results. In particular, our data do not contain information on aggressors. Therefore, we cannot establish whether a male-skewed classroom sex ratio instigates aggression primarily by male or female students. The literature on sex ratio effects on violent offending primarily focuses on male offenders [47, 48, 72, 73]. Yet, there is evidence that female violence also increases when sex ratios are male-skewed. In particular, research shows that girls’ delinquency is higher if they affiliate with male friends [74–76] and that having more male friends is associated with more antisocial behavior in girls [24, 77]. Thus, the lack of information on offenders prevents us from establishing whether the surplus injuries from aggression are actually driven by *male* aggression. Future studies should consider collecting details on the aggressor(s) to further explore the underlying mechanisms. Moreover, qualitative observational studies of peer dynamics or in-depth interviews with adolescents about their experiences in different sex ratio contexts are necessary to identify the particular mechanisms at play.

Despite these limitations, there are numerous strengths of our study including the nationwide scope and longitudinal nature of our data. The details on the classroom context and information on non-injured students helps us to elucidate that sex ratios are an important contextual factor for adolescent injury risks. On a broader theoretical level, our findings support concerns that male-skewed sex ratios are associated with an increase in aggression and violence [43, 47, 48, 54, 78, 79]. Additional research is needed that examines the underlying mechanisms how classroom sex ratios are associated with school injuries, specifically addressing the relation to aggression-related injuries. Nevertheless, this study provides a meaningful first step towards a more detailed exploration of school injury risks and classroom sex ratios as well as contextual effects in general.

## Conclusions

Our results reveal that male-skewed classroom sex ratios are associated with an increased risk for school injuries among adolescents, in particular school injuries resulting from aggression. Thus, our findings call for an integrated approach of prevention strategies at various levels to generate awareness regarding the potential health hazards of male-dominated adolescent classrooms. Schools and other stake holders should take into account the role of classroom sex compositions for adolescents’ risk behavior and aggression when designing schedules and planning interventions. For instance, funding agencies of prevention programs against school injuries and violence should consider prioritizing classes or schools with high shares of male students. Both risk-taking and aggression have an important signaling function since it confers status, particularly to adolescent males. Consequently, interventions should aim to provide specifically adolescents in male-dominated classrooms with alternative, safer forms of status attainment. Moreover, the present findings should inform future quantitative and qualitative investigations of classroom sex compositions and injury risks to better understand the underlying mechanisms and potential interactions with other factors.

## Supplementary Information

**Additional file 1.**

**Additional file 2.**

**Additional file 3.**

## Data Availability

The dataset analyzed during the current study is available in the OSF repository, https://osf.io/hax3u/?view_only=7ba31d3607e54875ad22a47bd7e6bee3
