# Supplementary material for: Adolescent school injuries and classroom sex compositions in German secondary schools
Source: BMC Public Health. 2022 Jan 10;22:62. doi: 10.1186/s12889-021-12370-8 (PMC8751068; doi:10.1186/s12889-021-12370-8)
Supplement: Supplementary file 1 — Additional file 1. [file 12889_2021_12370_MOESM1_ESM.pdf]

Adolescent school injuries and classroom sex compositions in German secondary schools

Supplementary Material 1

1 Figures

Figure S1: Class-level response rates and classroom shares of male students by wave, GUS data 2018-2019.

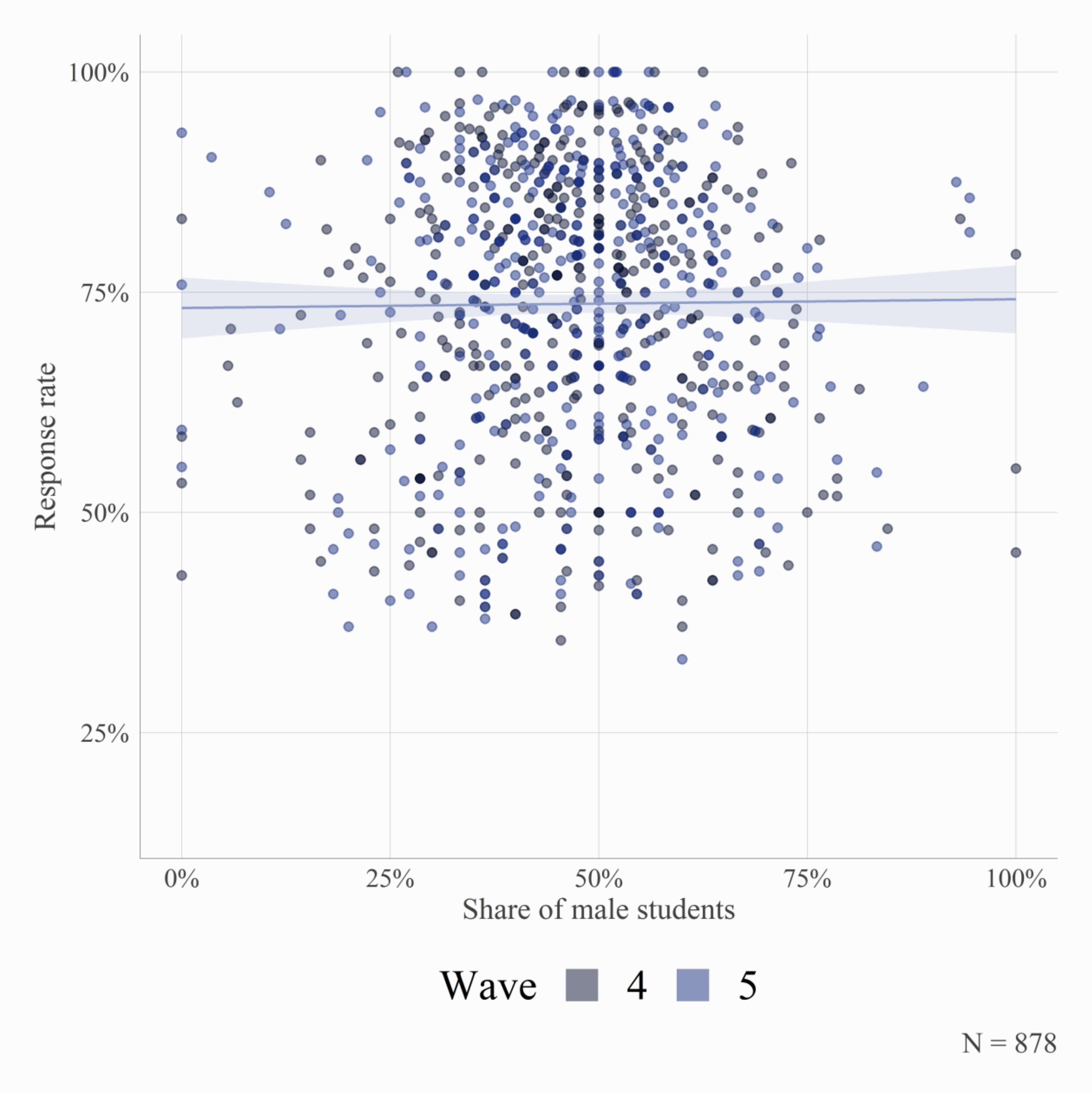

Figure S2: Adjusted predictions for school injury from bivariate multilevel logistic regression. Students from 8th and 9th grade, GUS data 2018-2019.

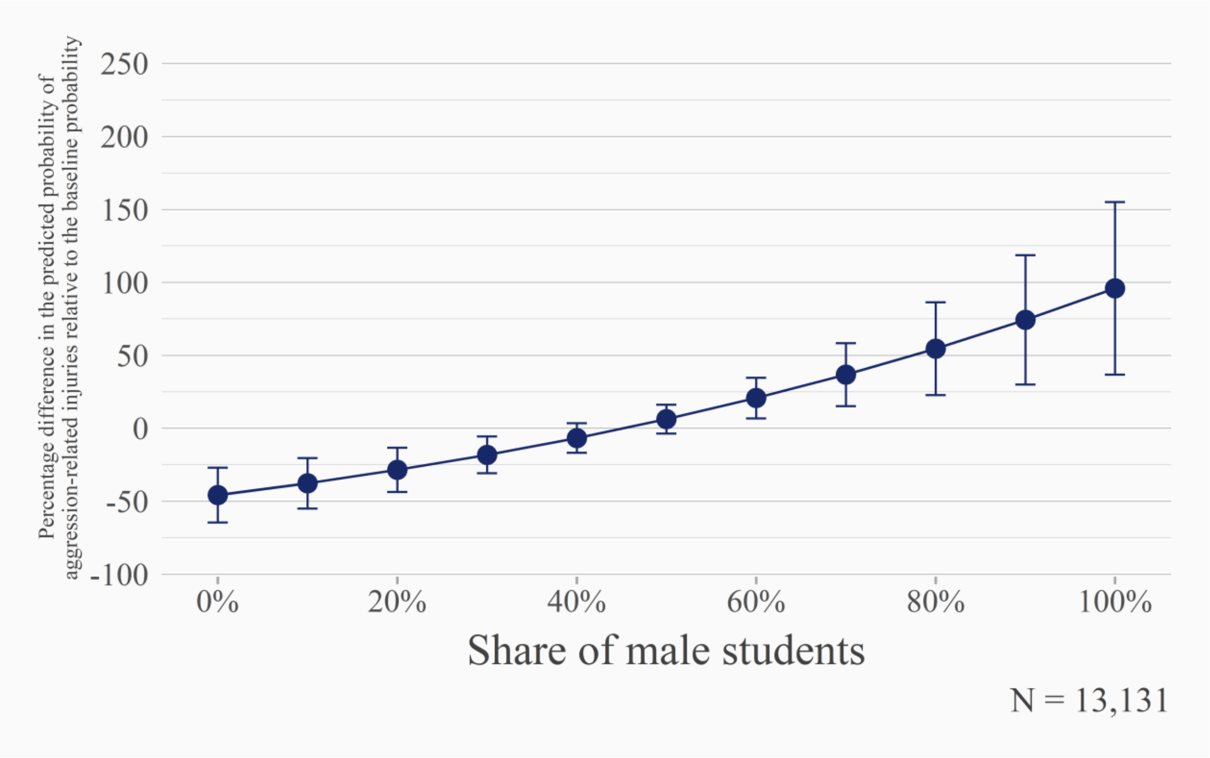

Figure S3: Adjusted predictions for school injury from multilevel logistic regression including an interaction term for sex. Students from 8th and 9th grade, GUS data 2018-2019.

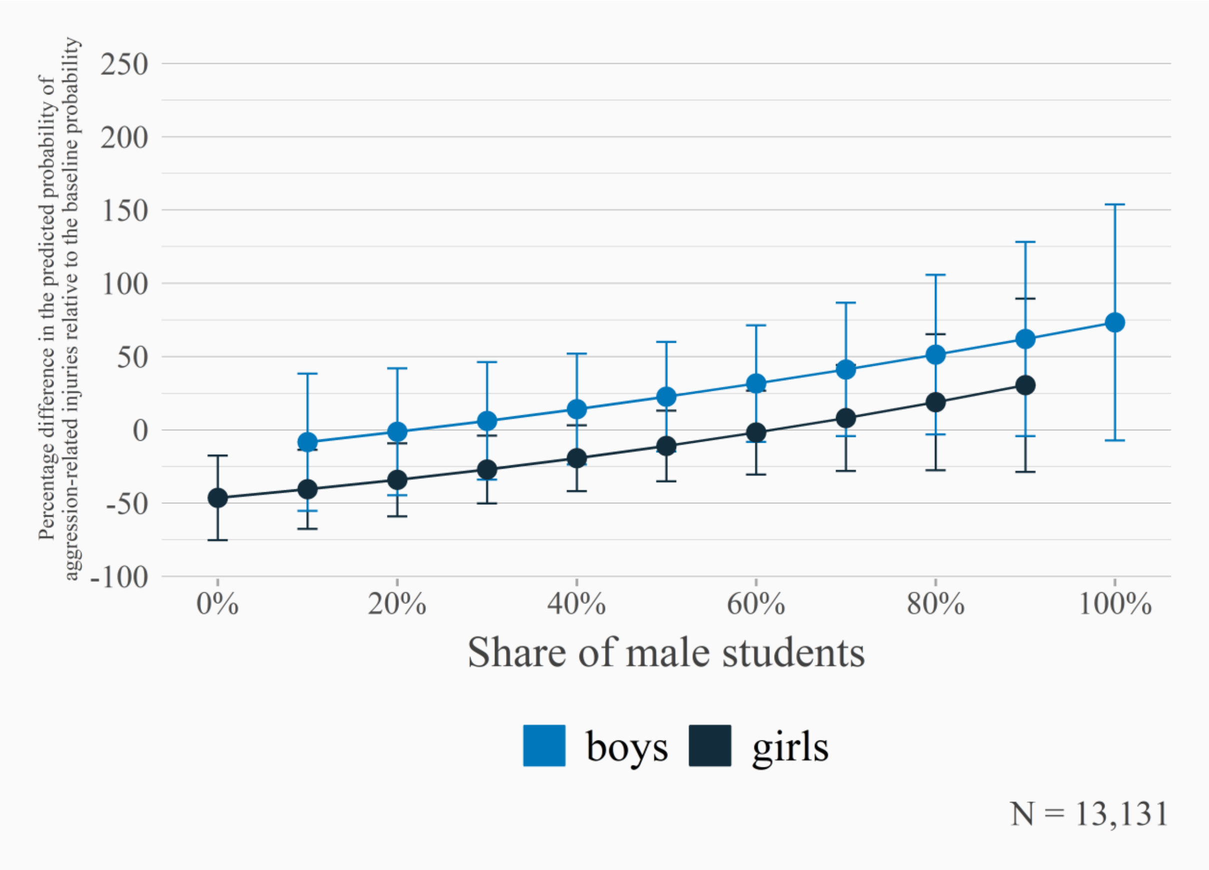

Figure S4: Adjusted predictions for school injury from aggression from bivariate multilevel logistic regression. Students from 8th and 9th grade, GUS data 2018-2019.

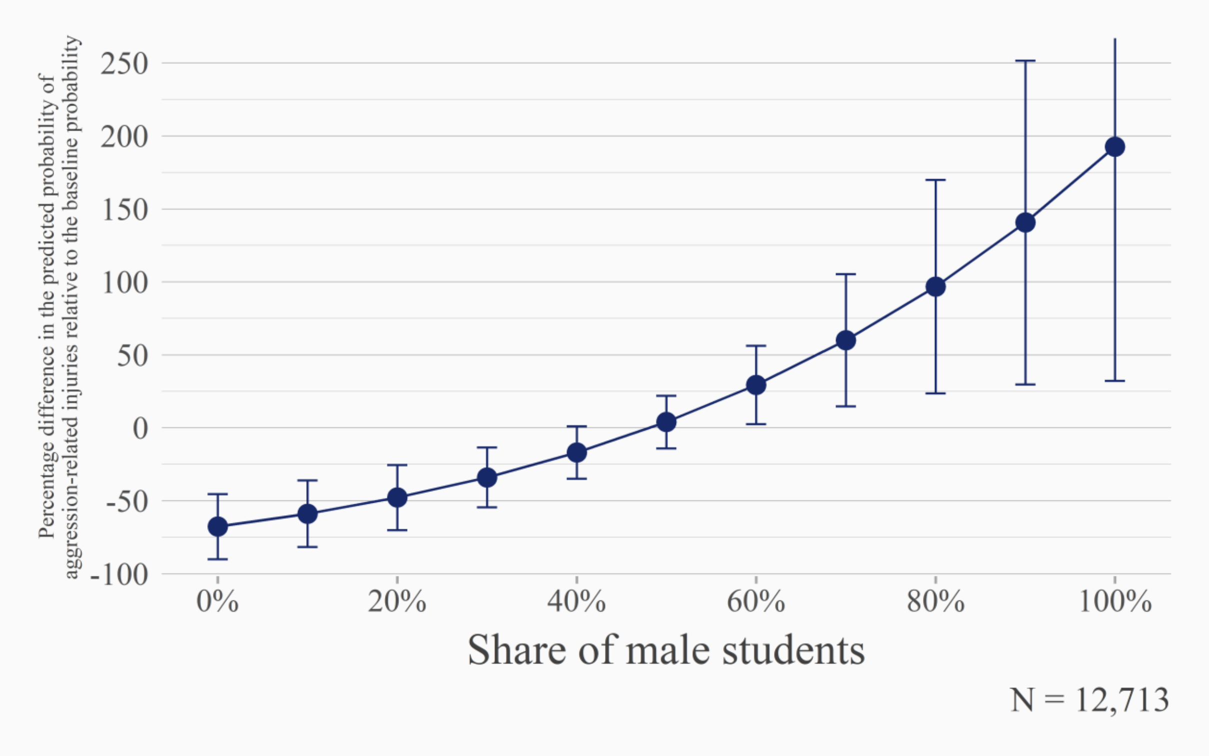

Figure S5: Adjusted predictions for school injury from aggression from multilevel logistic regression including an interaction term for sex. Students from 8th and 9th grade, GUS data 2018-2019.

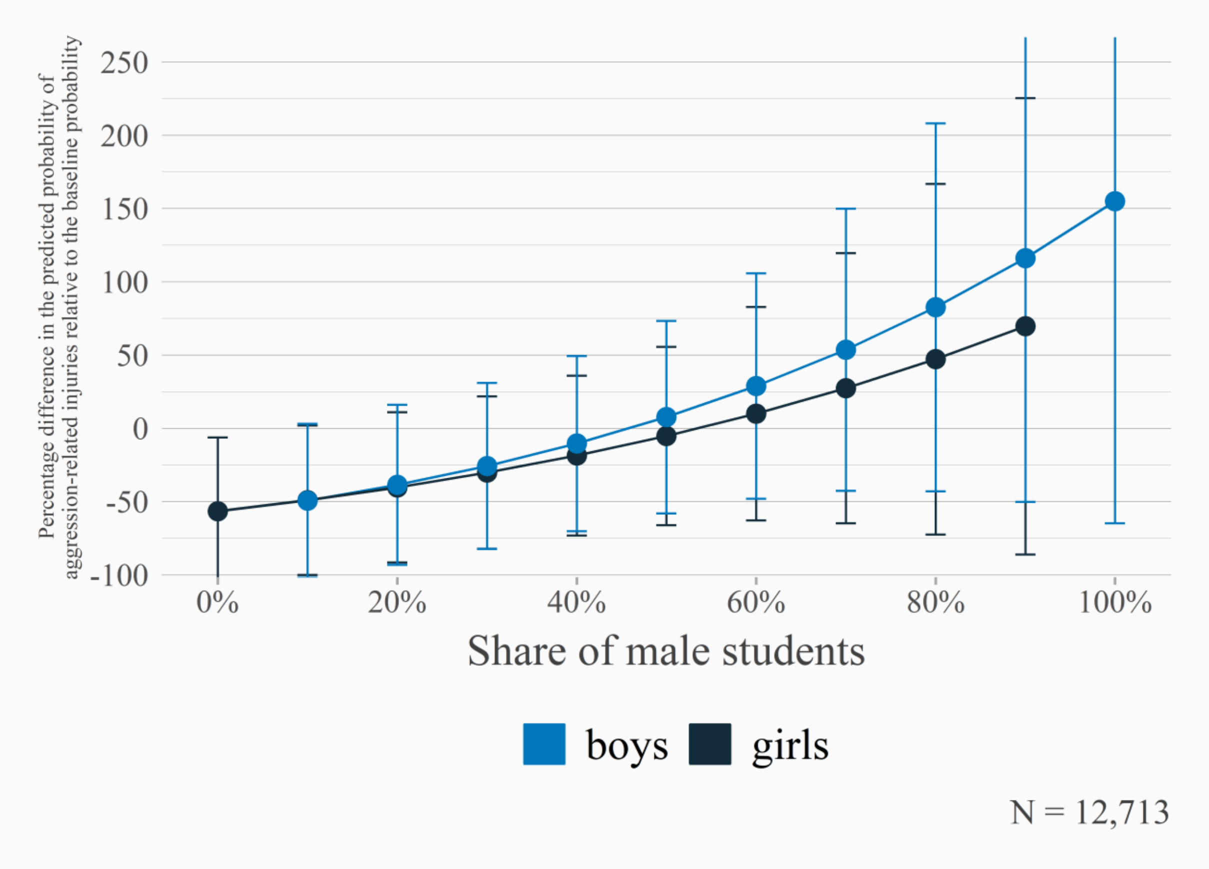

2 Tables

Table S1: Odds Ratios from bivariate multilevel logistic regression predicting the risk of injury on the school premises. Students from 8th and 9th grade, GUS data 2018-2019.

	Variable	OR	se	p	95% CI	
Fixed part	Classroom sex ratio (% male students)	1.017	0.0044	<.001	1.009	1.026
	Constant	0.0051	0.0018			
Random part	σ^2	0.321	0.128		0.147	0.701
	τ^2	3.423	0.735		2.247	5.215
LogLikelihood		-2,329.425				
N(students)		9,204				
N(class)		520				
N		13,131				

Models include individual- and aggregate-level variables as fixed effects and the respective class and person identifier as random effects. The terms σ^2 and τ^2 represent the variance between classes and individuals, respectively.

Table S2: Odds Ratios from multilevel logistic regression predicting the risk of injury on the school premises. Students from 8th and 9th grade, GUS data 2018-2019.

	Variable	OR	se	p	95% CI	
Fixed part	Classroom sex ratio (% male students)	1.012	0.0046	0.012	1.003	1.021
	Class size	0.996	0.02	0.853	0.958	1.036
	Class mean of family wealth	0.877	0.063	0.069	0.761	1.010
	Basic/intermediate secondary school	*Ref.*				
	Upper secondary school	0.621	0.099	0.003	0.455	0.847
	West German school	*Ref.*				
	East German school	1.056	0.195	0.768	0.735	1.517
	Urban school	*Ref.*				
	Rural school	0.908	0.143	0.539	0.667	1.236
	Sex: Female	*Ref.*				
	Sex: Male	1.793	0.224	<.001	1.402	2.291
	Migrant background: No	*Ref.*				
	Migrant background: Yes	1.368	0.179	0.017	1.059	1.767
	Mental health	0.521	0.039	<.001	0.449	0.604
	Family wealth	1.417	0.130	<.001	1.184	1.696
	8th grade	*Ref.*				
	9th grade	0.464	0.052	<.001	0.372	0.579
	Constant	0.02	0.015			
Random part	σ^2	0.258	0.123		0.101	0.658
	τ^2	3.414	0.751		2.218	5.256
LogLikelihood		-2,243.896				
N(students)		9,204				
N(class)		520				
N		13,131				

Models include individual- and aggregate-level variables as fixed effects and the respective class and the person identifier as random effects. The terms σ^2 and τ^2 represent the variance between classes and individuals, respectively.

Table S3: Odds Ratios from multilevel logistic regression predicting the risk of injury on the school premises. Students from 8th and 9th grade, GUS data 2018-2019.

	Variable	OR	se	p	95% CI	
Fixed part	Classroom sex ratio (% male students)	1.013	0.0063	0.037	1.001	1.026
	Male × Classroom SR	0.997	0.0084	0.713	0.981	1.013
	Class size	0.996	0.02	0.845	0.958	1.036
	Class mean of family wealth	0.878	0.063	0.072	0.763	1.012
	Basic/intermediate secondary school	*Ref.*				
	Upper secondary school	0.620	0.098	0.003	0.455	0.846
	West German school	*Ref.*				
	East German school	1.060	0.195	0.750	0.739	1.521
	Urban school	*Ref.*				
	Rural school	0.906	0.142	0.531	0.666	1.233
	Sex: Female	*Ref.*				
	Sex: Male	1.801	1.063	0.319	0.566	5.730
	Migrant background: No	*Ref.*				
	Migrant background: Yes	1.368	0.178	0.016	1.060	1.766
	Mental health	0.522	0.039	<.001	0.450	0.605
	Family wealth	1.414	0.130	<.001	1.181	1.692
	8th grade	*Ref.*				
	9th grade	0.465	0.052	<.001	0.373	0.580
	Constant	0.02	0.016			
Random part	σ^2	0.252	0.122		0.097	0.653
	τ^2	3.151	1.038		1.653	6.008
	υ^2	0.462	1.156		0.0034	62.442
LogLikelihood		-2,243.657				
N(students)		9,204				
N(class)		520				
N		13,131				

Models include individual- and aggregate-level variables as fixed effects and the respective class and person identifier as random effects. The terms σ^2 and τ^2 represent the variance between classes and individuals, respectively. The term υ^2 represents the random slope for individual sex. Including a random slope for the lower-level variable is recommended for cross-level interactions (see Methods section).

Table S4: Odds Ratios from bivariate multilevel logistic regression predicting the risk of injury from aggression on the school premises. Students from 8th and 9th grade, GUS data 2018-2019.

	Variable	OR	se	p	95% CI	
Fixed part	Classroom sex ratio (% male students)	1.028	0.0081	<.001	1.012	1.044
	Constant	0.00031	0.00022			
Random part	σ²	0.642	0.361		0.213	1.931
	τ²	5.018	1.310		3.008	8.371
LogLikelihood		-836.359				
N(students)		8,962				
N(class)		520				
N		12,713				

Models include individual- and aggregate-level variables as fixed effects and the respective class and person identifier as random effects. The terms σ² and τ² represent the variance between classes and individuals, respectively.

Table S5: Odds Ratios from multilevel logistic regression predicting the risk of injury from aggression on the school premises. Students from 8th and 9th grade, GUS data 2018-2019.

	Variable	OR	se	p	95% CI	
Fixed part	Classroom sex ratio (% male students)	1.022	0.0086	0.010	1.005	1.039
	Class size	1.043	0.038	0.252	0.970	1.121
	Class mean of family wealth	1.103	0.146	0.459	0.851	1.429
	Basic/intermediate secondary school	*Ref.*				
	Upper secondary school	0.315	0.094	<.001	0.176	0.566
	West German school	*Ref.*				
	East German school	1.700	0.553	0.103	0.898	3.216
	Urban school	*Ref.*				
	Rural school	0.697	0.197	0.202	0.401	1.213
	Sex: Female	*Ref.*				
	Sex: Male	2.038	0.475	0.002	1.291	3.218
	Migrant background: No	*Ref.*				
	Migrant background: Yes	1.697	0.411	0.029	1.056	2.726
	Mental health	0.560	0.075	<.001	0.431	0.727
	Family wealth	1.566	0.270	0.009	1.117	2.196
	8th grade	*Ref.*				
	9th grade	0.378	0.081	<.001	0.249	0.575
	Constant	0.00032	0.00048			
Random part	σ²	0.528	0.357		0.140	1.986
	τ²	5.146	1.413		3.004	8.815
LogLikelihood		-799.716				
N(students)		8,962				
N(class)		520				
N		12,713				

Models include individual- and aggregate-level variables as fixed effects and the respective class and the person identifier as random effects. The terms σ² and τ² represent the variance between classes and individuals, respectively.

Table S6: Odds Ratios from multilevel logistic regression predicting the risk of injury from aggression on the school premises. Students from 8th and 9th grade, GUS data 2018-2019.

	Variable	OR	se	p	95% CI	
Fixed part	Classroom sex ratio (% male students)	1.020	0.012	0.100	0.996	1.044
	Male × Classroom SR	1.004	0.016	0.784	0.973	1.036
	Class size	1.043	0.039	0.258	0.969	1.123
	Class mean of family wealth	1.098	0.147	0.487	0.844	1.427
	Basic/intermediate secondary school	*Ref.*				
	Upper secondary school	0.310	0.094	<.001	0.171	0.562
	West German school	*Ref.*				
	East German school	1.734	0.571	0.095	0.909	3.307
	Urban school	*Ref.*				
	Rural school	0.695	0.199	0.204	0.396	1.218
	Sex: Female	*Ref.*				
	Sex: Male	0.950	1.118	0.965	0.094	9.550
	Migrant background: No	*Ref.*				
	Migrant background: Yes	1.740	0.431	0.025	1.071	2.826
	Mental health	0.558	0.076	<.001	0.428	0.728
	Family wealth	1.586	0.280	0.009	1.122	2.241
	8th grade	*Ref.*				
	9th grade	0.373	0.081	<.001	0.244	0.571
	Constant	0.0004	0.00064			
Random part	σ^2	0.496	0.363		0.118	2.083
	τ^2	4.746	1.789		2.267	9.934
	υ^2	1.653	2.278		0.111	24.640
LogLikelihood		-799.299				
N(students)		8,962				
N(class)		520				
N		12,713				

Models include individual- and aggregate-level variables as fixed effects and the respective class and person identifier as random effects. The terms σ^2 and τ^2 represent the variance between classes and individuals, respectively. The term υ^2 represents the random slope for individual sex. Including a random slope for the lower-level variable is recommended for cross-level interactions (see Methods section).
