# Supplementary material for: Adolescent school injuries and classroom sex compositions in German secondary schools
Source: BMC Public Health. 2022 Jan 10;22:62. doi: 10.1186/s12889-021-12370-8 (PMC8751068; doi:10.1186/s12889-021-12370-8)
Supplement: Supplementary file 2 — Additional file 2. [file 12889_2021_12370_MOESM2_ESM.pdf]

Adolescent school injuries and classroom sex compositions in German secondary schools

Supplement 2: Results for different samples

Summaries for additional models to evaluate the sensitivity of our results to outliers in terms of the classroom sex ratio. In addition to the main sample, we analyzed three sample variants that either (1) exclude single-sex classes, (2) exclude classes with a sex ratio lower than 10% or above 90% male students, or (3) included only classes with a minimum class-level response rate of 80%.

S2.1: Bivariate models, any-cause injuries

Adjusted predictions for school injury from bivariate multilevel logistic regression models fitted to different sample configurations. Students from 8th and 9th grade, GUS data 2018-2019.

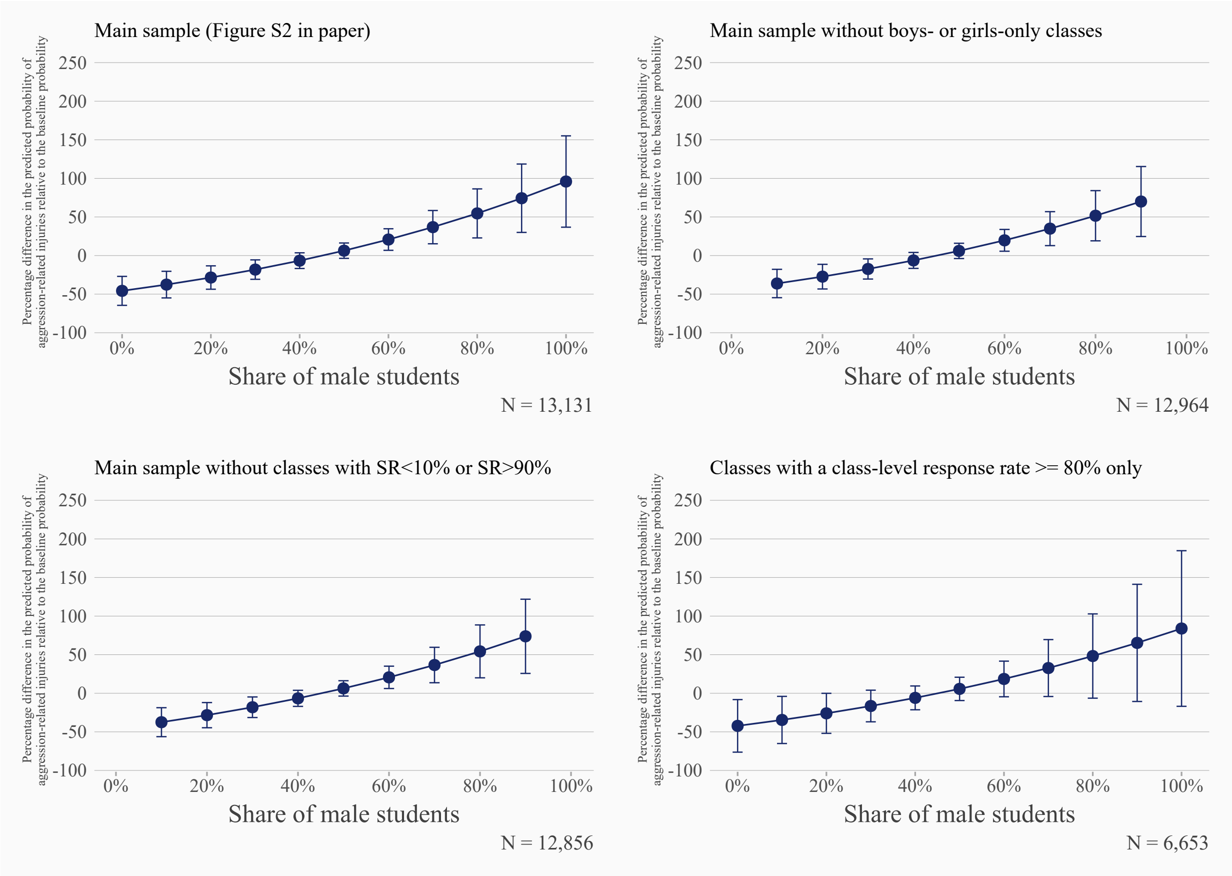

S2.2: Full models, any-cause injuries

Adjusted predictions for school injury from multilevel logistic regression models fitted to different sample configurations. Students from 8th and 9th grade, GUS data 2018-2019.

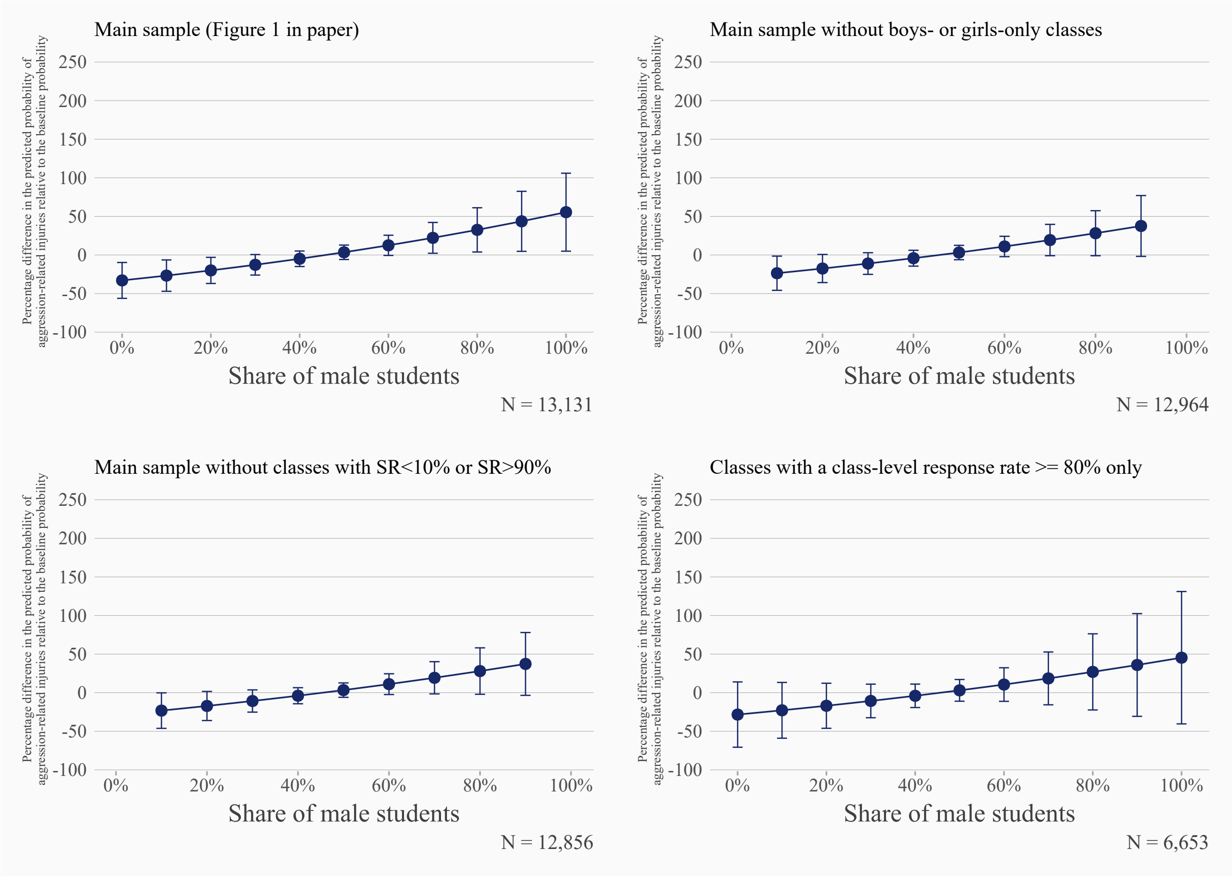

S2.3: Bivariate models, aggression-related injuries

Adjusted predictions for school injury from aggression from bivariate multilevel logistic regression models fitted to different sample configurations. Students from 8th and 9th grade, GUS data 2018-2019.

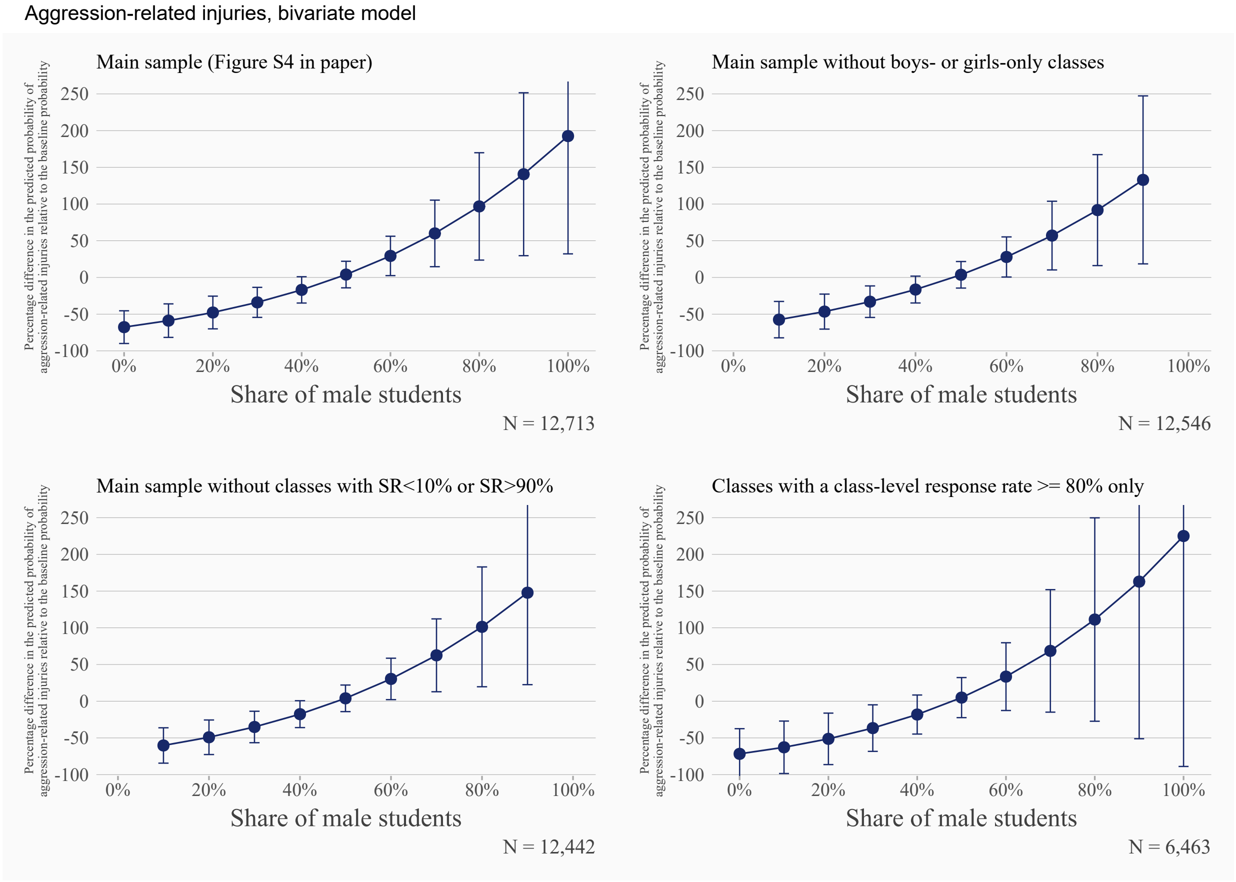

S2.4: Full models, aggression-related injuries

Adjusted predictions for school injury from aggression from multilevel logistic regression models fitted to different sample configurations. Students from 8th and 9th grade, GUS data 2018-2019.

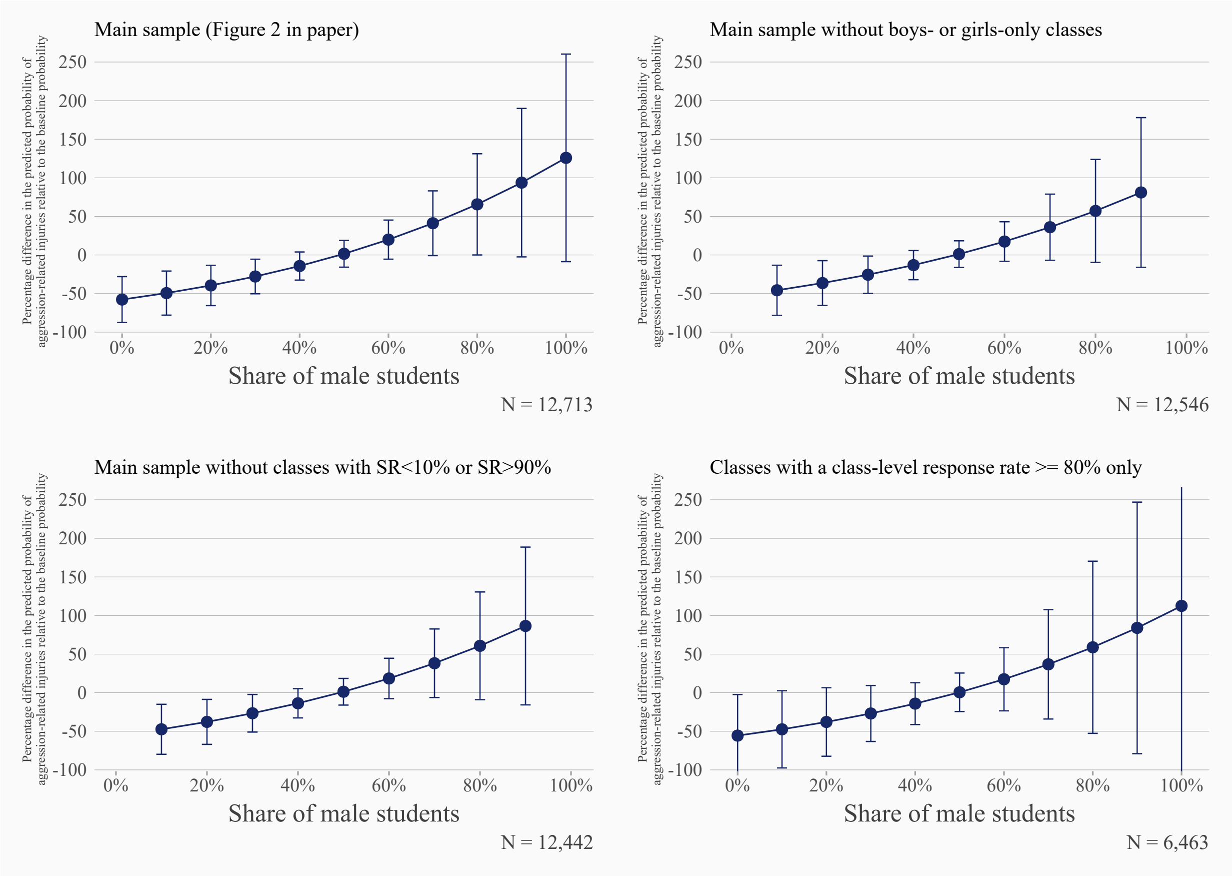

Table S2.1: Odds Ratios from bivariate multilevel logistic regression predicting the risk of injury on the school premises fitted to different sample variants. Students from 8th and 9th grade, GUS data 2018-2019.

	Variable	Main sample	Main sample without boys- and girls-only classes	Main sample without SR > 10% & SR < 90%	Classes with a class-level response rate >= 80% only
Fixed part	Classroom sex ratio (% male students)	1.017***	1.016***	1.017***	1.016*
		(0.0044)	(0.0046)	(0.0048)	(0.0081)
	Constant	0.0051***	0.0053***	0.0054***	0.0034***
		(0.0018)	(0.0019)	(0.0019)	(0.0021)
Random part	σ^2	0.321	0.308	0.295	0.517
		(0.128)	(0.127)	(0.124)	(0.248)
	τ^2	3.423	3.443	3.320	4.282
		(0.735)	(0.741)	(0.730)	(1.351)
LogLikelihood		-2,329.425	-2,321.001	-2,305.665	-1,101.008
N(students)		9,204	9,094	9,025	5,243
N(class)		520	514	510	271
N		13,131	12,964	12,856	6,653

Models include individual- and aggregate-level variables as fixed effects and the respective class and person identifier as random effects. The terms σ^2 and τ^2 represent the variance between classes and individuals, respectively.
Note: Standard errors in parentheses, + p<0.1; * p<0.05; ** p<0.01; *** p<0.001

Table S2.2: Odds Ratios from multilevel logistic regression models predicting the risk of injury on the school premises fitted to different sample variants. Students from 8th and 9th grade, GUS data 2018-2019.

	Variable	Main sample	Main sample without boys- and girls-only classes	Main sample without SR > 10% & SR < 90%	Classes with a class-level response rate >= 80% only
Fixed part	Classroom sex ratio (% male students)	1.012*	1.010*	1.010*	1.010
		(0.0046)	(0.0049)	(0.005)	(0.0085)
	Class size	0.996	1.000	1.000	0.983
		(0.020)	(0.020)	(0.020)	(0.029)
	Class mean of family wealth	0.877+	0.885+	0.881+	0.687**
		(0.063)	(0.064)	(0.064)	(0.082)
	Basic/intermediate secondary school	*Ref.*			
	Upper secondary school	0.621**	0.604**	0.610**	1.155
		(0.099)	(0.097)	(0.097)	(0.311)
	West German school	*Ref.*			
	East German school	1.056	1.036	1.031	0.639
		(0.195)	(0.191)	(0.189)	(0.192)
	Urban school	*Ref.*			
	Rural school	0.908	0.910	0.898	1.091
		(0.143)	(0.143)	(0.142)	(0.273)
	Sex: Female	*Ref.*			
	Sex: Male	1.793***	1.792***	1.773***	1.881**
		(0.224)	(0.224)	(0.220)	(0.366)
	Migrant background: No	*Ref.*			
	Migrant background: Yes	1.368*	1.360*	1.356*	0.862
		(0.179)	(0.178)	(0.177)	(0.178)
	Mental health	0.521***	0.518***	0.517***	0.440***
		(0.039)	(0.039)	(0.039)	(0.055)
	Family wealth	1.417***	1.414***	1.406***	1.473**
		(0.130)	(0.130)	(0.129)	(0.214)
	8th grade	*Ref.*			
	9th grade	0.464***	0.463***	0.468***	0.469***
		(0.052)	(0.052)	(0.053)	(0.084)
	Constant	0.02***	0.02***	0.021***	0.019**
		(0.015)	(0.015)	(0.016)	(0.023)
Random part	σ^2	0.258	0.242	0.231	0.361
		(0.123)	(0.122)	(0.120)	(0.225)
	τ^2	3.414	3.452	3.347	4.291
		(0.751)	(0.761)	(0.753)	(1.393)
LogLikelihood		-2,243.896	-2,234.611	-2,219.479	-1,053.768
N(students)		9,204	9,094	9,025	5,243
N(class)		520	514	510	271
N		13,131	12,964	12,856	6,653

Models include individual- and aggregate-level variables as fixed effects and the respective class and person identifier as random effects. The terms σ^2 and τ^2 represent the variance between classes and individuals, respectively.
Note: Standard errors in parentheses, + p<0.1; * p<0.05; ** p<0.01; *** p<0.001

Table S2.3: Odds Ratios from bivariate multilevel logistic regression predicting the risk of injury from aggression on the school premises fitted to different sample variants. Students from 8th and 9th grade, GUS data 2018-2019.

	Variable	Main sample	Main sample without boys- and girls-only classes	Main sample without SR > 10% & SR < 90%	Classes with a class-level response rate >= 80% only
Fixed part	Classroom sex ratio (% male students)	1.028***	1.027**	1.029**	1.033*
		(0.0081)	(0.0085)	(0.0089)	(0.016)
	Constant	0.00031***	0.00033***	0.0003***	0.00012***
		(0.00022)	(0.00024)	(0.00022)	(0.00016)
Random part	σ^2	0.642	0.637	0.617	1.169
		(0.361)	(0.360)	(0.356)	(0.771)
	τ^2	5.018	4.997	4.974	6.381
		(1.310)	(1.307)	(1.301)	(2.403)
LogLikelihood		-836.359	-831.291	-829.719	-407.209
N(students)		8,962	8,851	8,784	5,112
N(class)		520	514	510	271
N		12,713	12,546	12,442	6,463

Models include individual- and aggregate-level variables as fixed effects and the respective class and person identifier as random effects. The terms σ^2 and τ^2 represent the variance between classes and individuals, respectively.
Note: Standard errors in parentheses, + p<0.1; * p<0.05; ** p<0.01; *** p<0.001

Table S2.4: Odds Ratios from multilevel logistic regression predicting the risk of injury from aggression on the school premises fitted to different sample variants. Students from 8th and 9th grade, GUS data 2018-2019.

	Variable	Main sample	Main sample without boys- and girls-only classes	Main sample without SR > 10% & SR < 90%	Classes with a class-level response rate >= 80% only
Fixed part	Classroom sex ratio (% male students)	1.022*	1.020*	1.021*	1.021
		(0.0086)	(0.0092)	(0.0094)	(0.016)
	Class size	1.043	1.051	1.045	1.012
		(0.038)	(0.039)	(0.039)	(0.052)
	Class mean of family wealth	1.103	1.108	1.116	0.796
		(0.146)	(0.148)	(0.149)	(0.159)
	Basic/intermediate secondary school	*Ref.*			
	Upper secondary school	0.315***	0.308***	0.314***	0.551
		(0.094)	(0.093)	(0.094)	(0.256)
	West German school	*Ref.*			
	East German school	1.700	1.679	1.652	0.847
		(0.553)	(0.547)	(0.537)	(0.445)
	Urban school	*Ref.*			
	Rural school	0.697	0.723	0.696	0.525
		(0.197)	(0.206)	(0.199)	(0.216)
	Sex: Female	*Ref.*			
	Sex: Male	2.038**	2.041**	2.036**	2.051*
		(0.475)	(0.475)	(0.472)	(0.699)
	Migrant background: No	*Ref.*			
	Migrant background: Yes	1.697*	1.700*	1.701*	1.056
		(0.411)	(0.413)	(0.412)	(0.378)
	Mental health	0.560***	0.556***	0.556***	0.458***
		(0.075)	(0.074)	(0.074)	(0.093)
	Family wealth	1.566**	1.553*	1.553*	1.489
		(0.270)	(0.268)	(0.268)	(0.369)
	8th grade	*Ref.*			
	9th grade	0.378***	0.380***	0.382***	0.405**
		(0.081)	(0.081)	(0.082)	(0.127)
	Constant	0.00032***	0.0003***	0.00035***	0.0013**
		(0.00048)	(0.00046)	(0.00053)	(0.0028)
Random part	σ^2	0.528	0.525	0.515	0.697
		(0.357)	(0.357)	(0.354)	(0.608)
	τ^2	5.146	5.141	5.120	5.770
		(1.413)	(1.414)	(1.409)	(2.269)
LogLikelihood		-799.716	-794.184	-793.240	-389.470
N(students)		8,962	8,851	8,784	5,112
N(class)		520	514	510	271
N		12,713	12,546	12,442	6,463

Models include individual- and aggregate-level variables as fixed effects and the respective class and person identifier as random effects. The terms σ^2 and τ^2 represent the variance between classes and individuals, respectively.
Note: Standard errors in parentheses, + p<0.1; * p<0.05; ** p<0.01; *** p<0.001
